# Controlled heating between 100 and 500 degrees celsius improves enamel resistance to erosion in vitro

**DOI:** 10.1038/s41598-026-47191-5

**Published:** 2026-04-09

**Authors:** Richard J. Wierichs, Seyed Ahmad Banihashem Rad, Joel Glöckler, Marin D. Bilandzic, Stephanie A. Garofalo, Gabriella T. Schrotter, Samira H. Niemeyer, Guglielmo Campus, Hendrik Meyer-Lueckel, Marcella Esteves-Oliveira

**Affiliations:** 1 Department of Operative Dentistry and Periodontology, University Hospital Bonn, University of Bonn, Bonn, Germany; 2 Department of Restorative, Preventive and Pediatric Dentistry, University of Bern, Bern, Switzerland; 3 Department of Epidemiology and Health Promotion, New York University College of Dentistry, New York, NY USA; 4 Department of Conservative Dentistry, Periodontology and Endodontology, Faculty of Medicine, University of Tübingen, Tübingen, Germany; 5 Institute of Mineral Engineering (GHI), Chair of Glass and Glass-Ceramic, RWTH Aachen University, Aachen, Germany; 6 Department of Stomatology, School of Dentistry, University of São Paulo, São Paulo, Brazil; 7 Department of Cariology, Institute of Odontology, Sahlgrenska Academy at University of Gothenburg, Gothenburg, Sweden; 8 Department of Oral and Maxillofacial Sciences, Sapienza University of Rome, Rome, Italy; 9 Department of Cariology, Saveetha, Dental College and Hospitals, SIMATS, Chennai, India

**Keywords:** Oven, Laser, Erosive tooth wear, Prevention, Demineralization, Enamel, Wear, Fluoride, Tin, Dentistry, Disease prevention

## Abstract

This study aimed to investigate the effect of controlled heating at different temperatures on enamel erosion resistance, One hundred and twelve polished bovine enamel samples were randomly allocated to one of seven groups (n = 16): no treatment (negative control [C]); daily application of fluoride solution (500 ppm F^-^, 800 ppm Sn^2+^ as AmF/NaF/SnCl_2_, pH4.5, standard control [F]); and five oven-heated groups (100 °C, 200 °C, 300 °C, 400 °C, 500 °C). Heating in a dental oven was conducted atrates of 1 K/min, and each temperature level was maintained for 1 min. After surface treatment, all samples underwent a six-day erosive cycling protocol, with six daily immersions in citric acid (0.05 M, pH 2.3, 2 min) and storage in remineralization solution (≥ 1 h) between cycles. Enamel surface loss (µm) was measured at baseline and every second day (D2, D4, D6) using a 3D-laser-profilometer. Data were analyzed with two-way repeated measures ANOVA and *post-hoc* comparisons (p < 0.05). All heated groups (100–500 °C) and F significantly increased enamel erosion resistance compared to C (−24.6 ± 1.3 µm,p < 0.05). Furthermore, heating at 300 °C (−8.3 ± 2.7 µm), 400 °C (-5.6 ± 2.4 µm) and 500 °C (−3.2 ± 2.4 µm) resulted in significantly greater erosion resistance than F (−12.3 ± 2.3 µm, p = 0.02/ < 0.01), with 400 °C and 500 °C reducing enamel surface loss by 77 and 87%, respectively. Controlled heating significantly increased enamel erosion resistance, with 400 °C and 500 °C showing the highest protection. While oven heating is not clinically applicable, these findings contribute to understanding of CO₂ laser-induced erosion resistance. The significant increase in enamel erosion resistance following controlled heating suggests that structural modifications play a key role in erosion prevention. While oven heating provided standardized conditions, future research should explore whether similar protective effects can be achieved with clinically applicable CO₂-laser parameters to optimize non-invasive strategies for enamel preservation.

**Supplementary Information:**

The online version contains supplementary material available at 10.1038/s41598-026-47191-5.

## Introduction

Dental erosion, also known as erosive tooth wear, is an increasing concern both in younger and older age groups, making it an important oral health issue^1,2^. Despite public health efforts in many industrialized nations to improve oral health, its prevalence continues to rise, especially among younger adults^3^. However, reported prevalence rates are highly heterogeneous, ranging from 7.2 to 95%^4,5^. Estimates suggest that erosion affects 30–50% of primary teeth and 20–45% of permanent dentition^5^.

To effectively manage erosive tooth wear, it is crucial to identify and eliminate intrinsic and extrinsic acid sources, along with other risk factors as early as possible^6^. However, it may be difficult to eliminate the causative factors since they are often associated with habits or lifestyles and are influenced by nutritional, medical, psychological, and professional factors^7^. This enhances the need for effective preventive strategies to slow progression of tissue loss.

Among available preventive measures, polyvalent metal fluoride compounds, such as titanium tetrafluoride (TiF₄) and stannous fluoride (SnF₂), are highly effective in preventing enamel erosion and the tooth wear caused by chemical or mechanical factors^8,9^. Meta-analyses have shown that these compounds improve enamel resistance to erosive and abrasive forces, outperforming non-fluoride treatments^9,10^. The same positive anti-erosive effects for tin-containing solutions (AmF/NaF/SnCl_2_ mouthwash) and a TiF4/NaF solution have been shown recently in vivo^11,12^. However, the necessity of frequent application and patient adherence, limiting their practical application.

Given the advantages of prevention over (invasive) treatment, increasing tooth surface’s acid resistance is crucial for controlling dental erosion. While frequent fluoride and stannous treatments remain necessary to form protective layers or incorporate metal ions into the enamel^11,13^, no existing method provides a long-term, compliance-independent solution. This underscores the need for further investigation into more effective preventive strategies^14^.

One potential strategy involves controlled heating of enamel, which enhances mineralization and strengthens hydroxyapatite crystals, hence increasing its acid resistance ^15^. Heat accelerates calcium and phosphate ion deposition, increasing enamel density, reducing porosity, and improving structural alignment. However, excessive heat can cause pulpal damage, necessitating precise thermal control during dental procedures such as laser treatments^15–18^.

In particular, CO_2_ laser irradiation has been extensively studied to enhance enamel resistance to caries by improving thermal stability, chemical resistance, and reducing acid solubility^19,20^. For instance, CO₂ lasers with specific settings (*e.g.* 0.3 J/cm^2^, 5 μs pulses, 226 Hz) can improve caries resistance by up to 81% compared to negative controls, outperforming fluoride treatments (25%)^21^. Combining this specific parameter setting with a tin-containing fluoride solution has also been found to reduce enamel dissolution, reharden softened enamel, and nearly eliminate erosion both in vitro^22^ and in situ^23^. More recently an automatic laser scanning strategy has further optimized surface treatment, providing both consistency and improved erosion prevention^24^. However, the underlying mechanisms responsible for these protective effects are not yet fully understood.

Most theories attribute the erosion-protective effects of CO_2_ lasers to enamel temperature increase, especially above 100 °C^15–18^. However, the impact of systematically heating enamel at various temperatures on its resistance to acid-induced erosion has, to the best of the authors’ knowledge, not yet been investigated. Therefore, the present in vitro study was conducted to systematically evaluate the influence of controlled heating at temperatures ranging from 100 °C to 500 °C on enamel erosion resistance. Furthermore, histological and surface changes in enamel induced by different temperatures were assessed.

The null-hypothesis was that there would be no significant difference in erosive enamel surface loss between enamel heated from 100 °C to 500 °C and either the negative (no treatment) or the standard control (tin-containing fluoride solution). By clarifying the role of heat treatment in erosion prevention, the findings could aid in optimizing CO₂ laser parameters and developing tailored irradiation strategies for improved clinical applications.

## Materials and methods

### Samples preparation

**Fig. 1:**
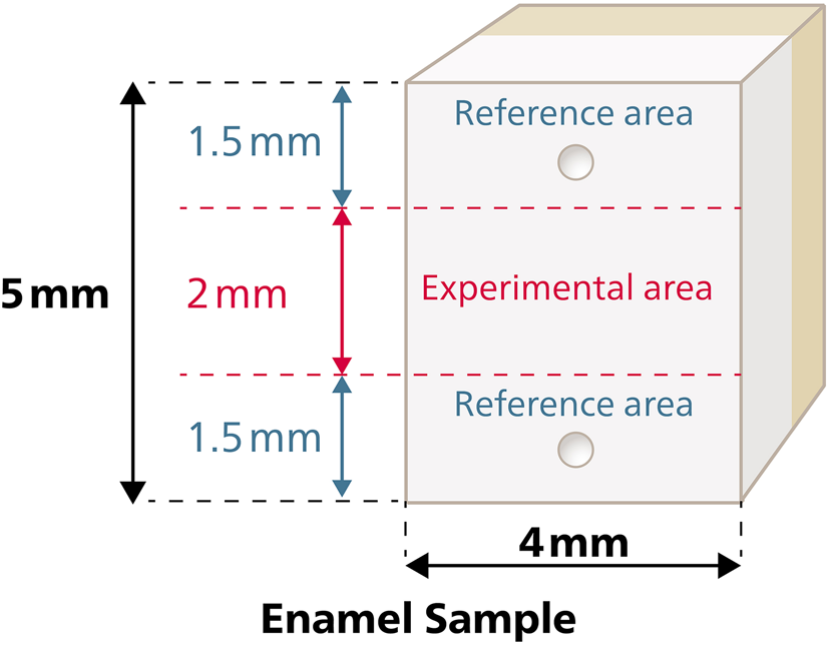
The experimental area had a width of 2 mm, designed to simulate the natural dimensions of both the cervical areas of the tooth as well as pit and fissure area of human molars, with potential future applications for erosion and caries prevention. Two reference areas (Ref.) with a width of 1.5 mm were preserved intact in all samples to facilitate the visualization and measurement of changes in surface profile and roughness.

One hundred and twelve enamel specimens (blocks of 4 × 5 x approximately. 2.5 mm) were prepared from sound bovine permanent incisors and were stored in a 0.1% thymol solution (Fig. 1). The teeth were examined by means of a stereomicroscope (4 × magnification) and only the ones presenting no cracks or structural defects were included in the study. The enamel surfaces were polished using P800, P1200, P2400 and P4000 silicon carbide grinding papers (Struers, Willich, Germany) on a rotating polishing machine (Exakt, Norderstedt, Germany) and immersed for 30 s in a sonication bath. A standardized removal of 400 ± 40 µm^25^ was controlled with a digital micrometer (Mitutoyo Deutschland, Neuss, Germany).

### Sample size

The sample size was calculated based on the results of a previous study with the same design for the erosive cycling^22^. According to that, mean enamel surface losses after 10 days of 27.2 ± (SD = 4.1) µm for the negative control [C] and 18.3 (SD = 4.4) µm for the laser group would be expected. To obtain a power of 80% a sample size of eight (enamel blocks) was calculated (two-sided, standard deviation = 6.1 and α = 0.05). In order to account for a possible different behavior of the laser groups here (new laser parameters), 16 samples per treatment per day were used in the experiments.

### Enamel surface treatment

Samples were randomly allocated to 7 groups (n = 16 profilometer) receiving different surface treatments:

- Negative control (**C**): no pre-treatment.
- standard control (**F):** Daily fluoride application using an anti-erosive solution (pH = 4.5) containing 500 ppm fluoride (125 ppm F^-^ as amine fluoride, 375 ppm F^-^ as sodium fluoride) and 800 ppm of stannous as stannous chloride (Elmex Erosionsschutz, Gaba, Lörrach, Germany). Specimens were immersed at the beginning of each cycling day in this solution for 30 s, under agitation on an orbital shaker (Orbit, Labnet, Edison, USA).
- Heated groups (**100, 200, 300, 400, 500**): Whole samples were oven-heated to 100 °C, 200 °C, 300 °C, 400 °C, or 500 °C.

A pilot test was conducted to establish the most appropriate heating parameters, evaluating two heating rates (1 K/min and 5 K/min), as well as 3 holding times at the final temperature (1, 3 and 5 min). The results showed that heating the samples at the lower heating rate and the lowest holding time for the final temperature caused substantially less sample breakdowns (Supplementary Material Figs. 1, 2).

**Fig. 2:**
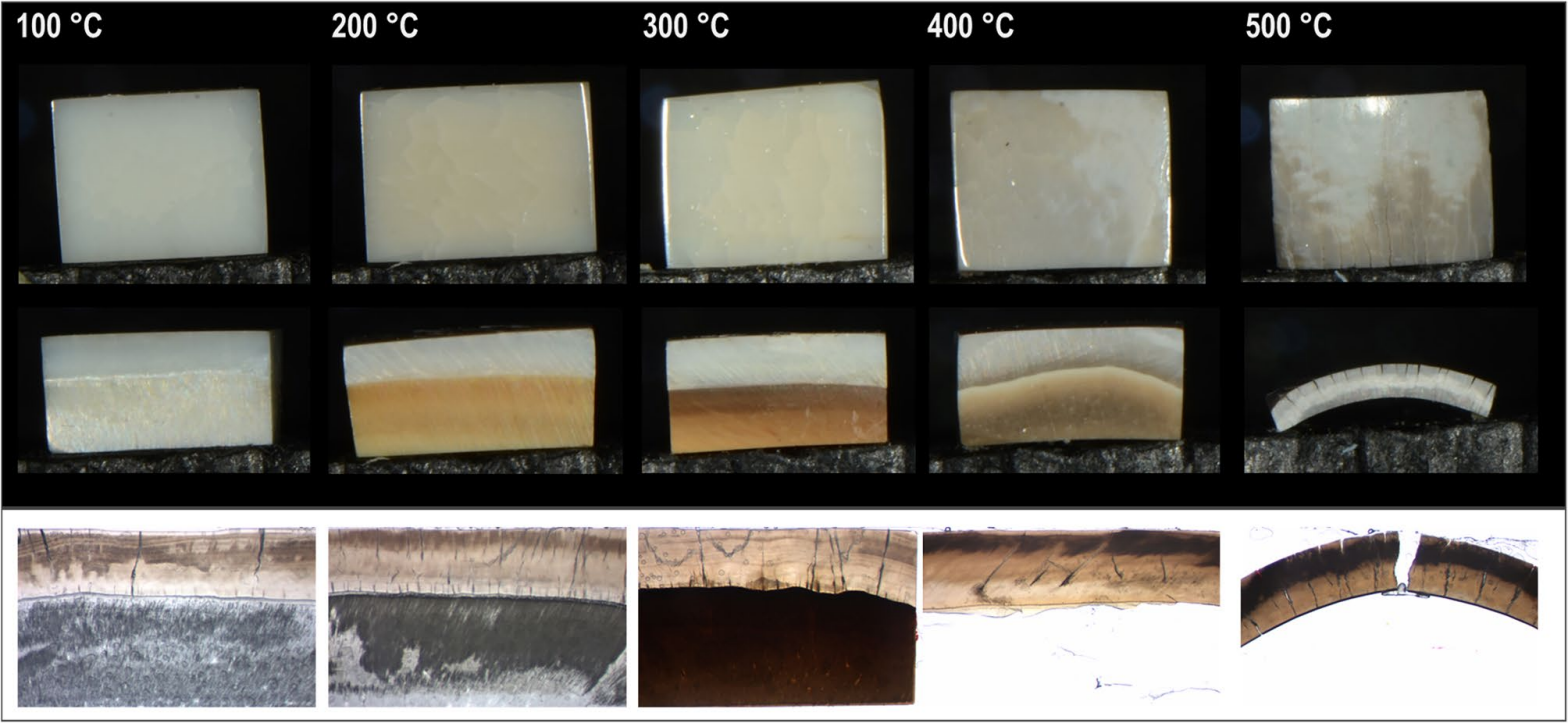
Representative images of all oven-heated groups (100–500 °C). The upper row shows the enamel surface appearance, the middle row displays the lateral side of the sample, and the bottom row provides histological views of the cross-Sects. (100 ± 10 µm, bottom line). Note the progressive color changes in dentin (yellow to dark brown) and enamel curvature at higher temperatures.

Before heating, the samples were dried with cellulose cloths and placed on a base made of cordierite stone (high temperature resistance). Based on the pilot test, samples in all oven-heated groups were heated only once, prior pH-cycling, in a dental oven (type 312,476, Therm-AIX, Aachen). Heating started from room temperature (23 °C) and followed a rate of 1 K/min for both heating and cooling, with a final holding time of 1 min at the designated temperature. After heating care was taken to ensure that there was no residue of the cellulose wipes on the samples. The specimens were arranged 4 × 4 and placed at a constant distance of ca. 10 mm from each other. After heating, all specimens were rehydrated by storing them in a fully humidified environment (0.1% thymol solution) at room temperature for one week prior to the erosive cycling.

### Histology

After oven heating, representative samples from each experimental group (n = 3) were randomly selected for histological evaluation. Samples were sectioned in the center with a diamond saw and one half of each sample was serially dehydrated through a graded ethanol series (70, 80, 90, 96 and 100%) to remove moisture content. Subsequently samples were embedded in acrylic resin (Technovit 7200 VLC, Kulzer, Germany) to preserve the specimen structure for histological analysis. A two stage infiltration process was used, firstly by immersing the teeth in a mixture of ethanol and methacrylate monomer (Technovit1 7200 VLC, Heraeus-Kulzer, Wehrheim, Germany) in​ proportions of 1:1 for 2 h, followed by a pure monomer (Technovit1 7200 VLC) for 2 h. ​The acrylic resin infiltrated samples was then polymerised using visible light from fluorescent tubes that replicate daylight (5600 K, Histolux, Kulzer Exakt, Wehrheim, Germany) for 10 h. ​Following resin polymerization, the embedded samples were carefully polished to a final thickness of 100 ± 10 µm to allow optimal light transmission for microscopic evaluation. Sections were mechanically polished with silicon carbide papers under constant irrigation. These histological sections were then examined under a transmitted-light microscope equipped with a high-resolution digital camera (DM-R-HR, Leica, Germany)^21^.

### Erosive cycling

After surface treatment and before the erosive cycling removable acrylic-coated tapes (tesa 4651 black, Hamburg, Germany) of 1.5 mm width were fixed at the borders of the samples in order to preserve two reference areas for the profilometric analysis. This procedure was tested previously and proved not to cause any significant surface loss at the reference area^22^. Each two samples were then fixed on custom-made plastic slides (dimensions 8 × 2 cm) to enable the later attachment to a rack allowing simultaneous insertion of all samples into falcon tubes (2 samples/tube, ca. 2.8 ml solution/mm^2^ of exposed enamel). Samples were stored in a fully humidified environment at all times.

All specimens were demineralized 6 times daily for 2 min with 0.05 M citric acid (Merck), pH 2.3 as described previously^26^. Citric acid (pH 2.3) was selected as it closely mimics dietary acid exposure in an erosive environment .Between demineralization periods, there was an interval of at least 1 h in which the specimens were stored in a supersaturated mineral solution (4.08 mM H_3_PO_4_, 20.10 mM KCl, 11.90 mM Na_2_CO_3_ and 1.98 mM CaCl_2_, pH 6.5; chemicals from Merck)^27^. By means of a special rack, specimens of all groups could be moved simultaneously in and out of the falcon tubes, each containing 45 ml of the de- and remineralization solutions. Each rack supported 8 falcon tubes, and a sample holder (plastic slide) for 2 samples was fixed in each tube. Before transfer to the next solution, specimens were rinsed with tap water. During the whole pH-cycling period, samples were stored at room temperature and under agitation on an orbital shaker at 50 rpm. Overnight, specimens were kept in the remineralization solution and all solutions were renewed daily.

### Erosion – Enamel surface loss

After treatment (baseline) and at the end of every second cycling days (D2, D4, D6) enamel surface loss (µm) was measured using a 3D laser profilometer (VK-X100 Keyence, 20 × Objective, Neu-Isenburg, Germany). Before the measurements, the tape was removed from the reference areas and replaced afterwards. After tape removal, the reference areas were carefully cleaned with cotton pellets and checked under the laser microscope, to make sure that they were free of any adhesive remnants. To assure that the measurement of the enamel surface loss was at the same position as at the start of the experiment, two marks were engraved on the reference area of each sample using a fissure bur (Hager & Meisinger, Neuss, Germany), one at the left and one at the right side of the experimental area. For each specimen a 4400 × 530 µm wide rectangle was scanned, beginning in the reference area at one side, then going through the experimental area and ending in the reference area on the other side. With the corresponding analysis software (VK Analysis Application, Keyence, Neu-Isenburg, Germany) the scanned surface was adjusted so that the reference areas of both sides were exactly at the same height, using the tilt correction function for 3 points. After that, a horizontal line scan connecting the two bur grooves and going through the center of the lesion was used to conduct measurements. Along this line scan the average height difference between the reference areas and the experimental area was determined. Each day, the line was relocated at the same position to ensure the correct measurement of any height alteration in the experimental area. For the 400 °C and 500 °C groups a special measuring tool from the microscope software was used to compensate for the curvature of the samples. Directly at the beginning of the measurements tilt correction function was set to curved surfaces profile. Repeated measurements (n = 5) of one randomly selected specimen gave a standard deviation of 0.1 µm and the maximum height resolution of the optical profilometer was 5 nm.

### Scanning electron microscopy—SEM

In order to verify the effects obtained by profilometry and observe the effect of cycling and of the different treatments on the enamel surface and subsurface, morphological investigations were performed. After the erosive cycling representative samples of each group (n = 3) were randomly selected and sectioned with a band saw through the centre of the lesion in order to allow a cross-sectional view.

The samples were then serially dehydrated in alcohol, immersed in hexamethyldisilazane (HMDS) for further dehydration, covered with a thin gold layer and examined under an environmental scanning electron microscope (ESEM XL30 Field Emission Gun, Phillips, Eindhoven, Netherlands). The images were obtained using a GSED (gaseous secondary electron detector) detector with the sample’s chamber pressure at around 1 mbar, using an accelerating voltage of 10 kV.

### Statistical analysis

The data has been entered into Microsoft Excel 2016 (Microsoft Corporation, Washington, DC, USA) and analyzed with IBM SPSS Statistics (Version 27, IBM Corp., Armonk, NY, USA). All statistical tests were two-tailed, and the significance level was set at α = 0.05. Prior to conducting the analyses, the assumptions of normality and homogeneity of variances were evaluated using the Shapiro–Wilk test and Levene’s test, respectively**.**

Changes in enamel surface loss over time and between temperature groups were evaluated using a two-way repeated-measures ANOVA, with time (days) as the within-subject factor and treatment as the between-subject factor. Statistical reporting follows the SAMPL guidelines^28^ and current recommendations emphasizing estimation and effect sizes over dichotomous significance testing^29^. Accordingly, ANOVA outcomes are reported with F-statistics, exact p-values, and partial η^2^ as the appropriate effect size measure for repeated-measures designs. SPSS provides effect sizes only for the main ANOVA factors (Time, Treatment, and Time × Treatment). Planned comparisons between each heated group and the negative control were interpreted without multiplicity correction, following the rationale that adjustment is not required for pre‑specified comparisons^30^.

## Results

### Histology

In general, there was a correlation between rising temperatures and histological changes, with higher temperature leading to more pronounced histological alterations (Fig. 2). At 100 °C, dentin showed no noticeable changes, though some indication of micro cracks were visible at the enamel surface. At 200 °C, dentin became more yellow, progression to light brown at 300 °C and dark brown with some detachment from enamel at 400 °C. At 500 °C, enamel became very curved, with complete separation from dentin. Furthermore, samples exhibited an increase in transverse cracks on the enamel surface with rising temperatures.

#### Erosion

**Fig. 3:**
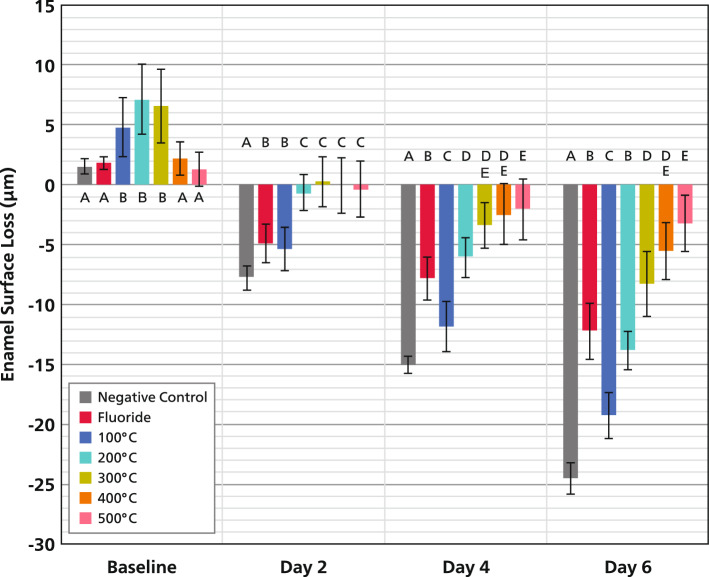
Mean enamel surface loss (µm) and standard deviations of all groups at the different examining days. Different letters indicate statistically significant differences between treatments within one time point (*p* < 0.05).

**Fig. 4:**
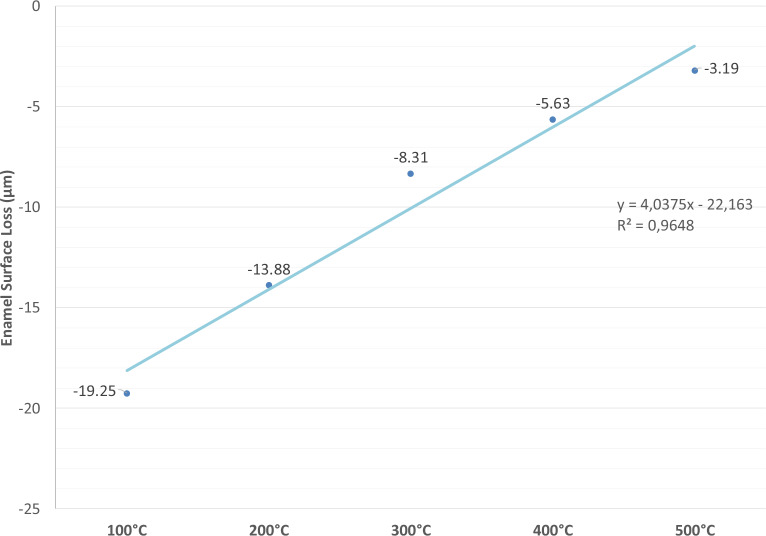
Mean enamel surface loss plotted over the different temperature levels, showing a strong direct linear correlation (R^2^ = 0.96). The higher the temperature in the range from 100 to 500 °C the higher the increase of erosion resistance (less enamel surface loss).

Enamel surface loss at baseline and of every second day is shown in Fig. 3 and Fig. 4. From day 2 onward, all heat-treated groups presented significant lower surface loss than the negative control [C] (-24.6 ± 1.4 µm, p < 0.05). However, on day 6, only 300 °C (-8.3 ± 2.8 µm), 400 °C (-5.6 ± 2.4 µm) and 500 °C (-3.2 ± 2.5 µm) groups showed significantly decreased enamel surface loss when compared to both the C and F, receiving daily tin-containing fluoride treatment (-12.3 ± 2.4 µm, p < 0.05).

The least anti-erosive effect was shown by the 100 °C group (-19.3 ± 1.9 µm), while the highest percentual reduction relative to C was observed by the 400 °C and 500 °C groups (77% and 87% respectively). By comparison fluoride treatment reduced enamel surface loss by 50%.

Statistical reporting follows the SAMPL guidelines^28^ and current recommendations emphasizing estimation and effect sizes over dichotomous significance testing^29^. The repeated-measures ANOVA revealed significant main effects for time and treatment, as well as a significant time** × **treatment interaction. Effect size analysis demonstrated very large effects for all primary factors (Table 1). Time (Days) accounted for nearly all explained variance in erosive progression (F(3,45) = 1238.72, p < 0.001, partial η^2^ = 0.988). Treatment showed a substantial effect (F(6,90) = 14.09, p < 0.001, partial η^2^ = 0.484). The time** × **treatment interaction was also large (F(18,270) = 37.25, p < 0.001, partial η^2^ = 0.713), indicating that the influence of heating varied systematically across the erosive cycling period. These findings confirm the robustness of the treatment effects and support the interpretation that controlled heating markedly modifies the temporal pattern of enamel erosion.

**Table 1:** Summary of effect sizes of the factors under analysis.

Effect	*F*-Statistic	p-value	partial ${{\boldsymbol{\eta}}}^{2}$
Time (Days)	*F(3,45)* = ***1238.72***	< 0.001	0.988
Treatment	*F(6,90)* = ***14.09***	< 0.001	0.484
Time x Treatment	*F(18,270)* = ***37.25***	< 0.001	0.713

**Fig. 5:**
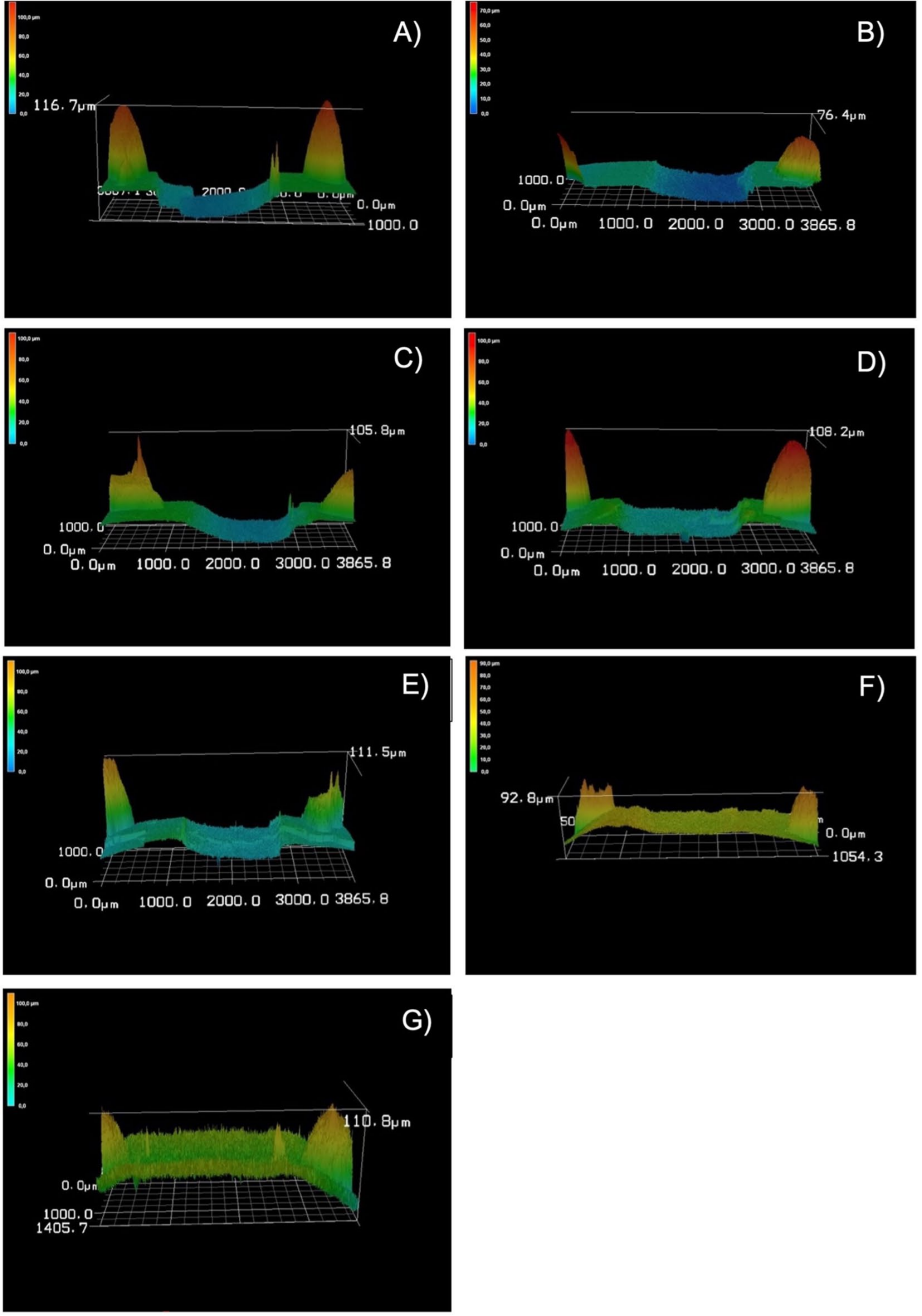
Representative images obtained with the 3D-laser microscope of all groups after the erosive cycling. The experimental areas are located at the center of the samples, while the reference areas are on the left and right sides. The peaks in the reference areas represent the bur grooves, which were used to ensure profilometric measurements were consistently taken from the same position within each sample. The highest enamel surface loss was observed in the negative control group (**A**), while the lowest loss was observed in the 400 °C (**F**) and 500 °C (**G**) groups. The positive standard control group exhibited a reduced but still measurable surface loss. Description: (**A**) negative control group, (**B**) standard control group (tin-containing fluoride), (**C**) 100 °C, (**D**) 200 °C, (**E**) 300 °C, (**F**) 400 °C, (**G**) 500 °C.

A strong linear relationship was observed between temperature and the magnitude of the preventive effect at Day 6 (n = 7 group means; R^2^ = 0.96; slope p = 0.022; 95% CI for the slope: [0.012–0.057]; Fig. 4). The higher the temperature in the range from 100 to 500 °C the higher the erosion resistance observed (less surface loss). Representative 3D images obtained with the laser microscope for all groups are shown in Fig. 5.

### Scanning electron microscopy—SEM

**Fig. 6:**
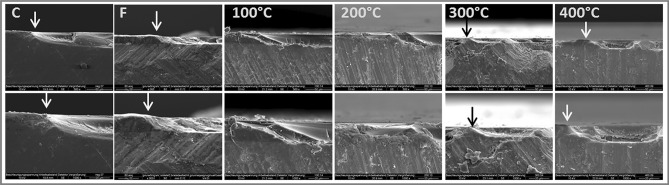
Representative cross-sectional images obtained by scanning electron microscopic of all groups (excluding 500 °C, which was lost due to severe curvature) after treatments and at the end of erosive cycling. Arrows show the point where the transition between reference area (left) and erosive lesion (right). For control samples a clear erosive lesion is seen, and for 300 °C and 400 °C lesions are very swallow. Magnifications × 500 (upper line) and × 1000 (bottom line).

Due to strong curvature, the enamel samples of the 500 °C group could not be analyzed. In all other groups, the samples showed enamel surface loss due to erosive cycling (Fig. 6). The C group presented the deepest erosion lesions, while erosive surface loss of F, 300 °C and 400 °C exhibited visibly shallower lesions. Some curvature of the enamel surface can also be observed at 400 °C.

## Discussion

The present in vitro study investigated the effect of controlled heating from 100 °C to 500 °C on enamel erosion resistance. All heat-treated groups exhibited significantly higher erosion resistance compared to the negative control. Notably, enamel heated to 300 °C, 400 °C, and 500 °C showed significantly less surface loss than both the negative control and the standard control group treated with tin-containing fluoride, the current gold standard for erosion prevention. Moreover, a correlation was observed between temperature increase from 100 to 500 °C and decreasing surface loss. Thus, the null-hypothesis had to be rejected.

Morphological changes increased with rising temperatures, affecting both enamel and dentin. Interestingly, these effects were more pronounced in dentin than in enamel. Between 200 °C and 400 °C, dentin color progressively changed from intensive yellow to light brown and finally dark brown. At 400 °C and 500 °C there was a complete detachment between dentin an enamel. At the surface of enamel though, more intensive changes were observed at 400 °C and 500 °C, with enamel experiencing a complete curvature at the highest temperatures. These morphological alterations were expected and certainly much more pronounced than those typically seen after laser irradiation. Several factors contribute to this difference. First, in the present study, the whole enamel-dentin samples were heated from all sides, whereas laser irradiation primarily affects the upper enamel surface. Second, the heating in the oven lasted 600 to 800 min (10–13 h) including heating and cooling processes, even though the highest temperatures were maintained only for 1 min. This is immensely longer than the heating caused by laser irradiation protocols for preventive treatment of enamel, which are delivered in some fractions of a second (dozens of µs) and whole treatment takes 10–19 s^24^. Third, in a clinical scenario, pulpal blood flow would help dissipate heat from dentin, a factor not replicated in this in vitro setup^31^. Thus, heating dental samples in an oven is clearly an extremely exaggerated simulation of the influence of high temperatures on enamel and dentin structures. Nevertheless, this approach provided standardized conditions for systematic analyses and future comparisons with laser irradiation effects. Therefore, future studies should investigate whether the observed linear relationship between temperature increase, and erosions resistance also applies to systematically controlled laser-generated heating of enamel surface.

The number of studies similar to the present study is very limited. Therefore, direct comparisons with existing research are challenging. However, one study^32^ analyzed the thermal response of teeth from various age groups to temperatures of 400 °C, 700 °C, and 1000 °C, finding that color changes in teeth could indicate thermal exposure, with deciduous teeth exhibiting less damage compared to permanent teeth. Another study^33^ assessed the temperature dependence of specific heat in human enamel and dentin, revealing that specific heat increases with temperature, ranging from 709 to 921 J·kg⁻^1^·K⁻^1^ between 20 °C and 70 °C. This finding suggests that enamel’s ability to absorb and retain heat improves with increasing temperatures, influencing its thermal behavior and resistance to damage. These insights support the hypothesis that temperature-induced modifications in dental tissues could enhance erosion resistance and overall structural durability.

Unlike conventional oven heating, CO₂ laser irradiation offers a transformative advantage: highly localized, temporally precise energy delivery. Pulsed laser emissions, lasting micro- to nanoseconds can elevate enamel surface temperatures to 300–500 °C almost instantaneously, yet remain below the thermal relaxation time (~ 90 µs at 10.6 µm), confining heat to the irradiated volume and preventing pulpal injury^34^. This enables targeted morphological and structural changes, such as carbonate loss and microstructural reorganization, which enhance enamel acid resistance while maintaining the morphological integrity of enamel and dentin. As demonstrated by Zuerlein et al. (1999)^34^, the depth and nature of enamel modification are strongly governed by pulse duration and absorption characteristics. Notably, the 10.6 µm CO₂ laser wavelength exhibits one of the highest absorption coefficients in enamel (825 cm⁻^1^), with an absorption depth of merely 12 µm—making it exceptionally well-suited for surface-selective heating. This precise control enables us to replicate the beneficial effects of high-temperature treatment while minimizing thermal risk to underlying tissues^35,36^. By leveraging a deep understanding of enamel’s optical and thermal properties, we can now engineer laser energy delivery conditions that selectively heat only a few dozen micrometers of the enamel surface to optimal temperatures for enhancing acid resistance. This approach goes far beyond simply avoiding pulpal injury^37^,it represents a paradigm shift toward precision-guided therapeutic modification of dental hard tissues.

Another study^38^ was conducted to assess the impact of oven heating and Er,Cr:YSGG laser irradiation on dental enamel to enhance its resistance to demineralization. Bovine dental enamel samples were thermally treated at temperatures ranging from 200 °C to 1000 °C and additionally exposed to laser irradiation with energy densities of 7.53, 10.95, and 13.74 J/cm^2^. The surface temperature during laser irradiation was assessed using infrared thermography, while Fourier Transform Infrared Spectroscopy (FTIR) analyzed alterations in carbonate, adsorbed water, and hydroxyl content. The study revealed that laser irradiation resulted in a reduction in carbonate content when compared to untreated enamel. Additionally, higher temperatures resulted in the removal of adsorbed H₂O and structural OH⁻. The modifications in enamel properties, such as solubility and resistance to demineralization, were more pronounced in laser-irradiated samples than those subjected to oven heating.

Consequently, a key question arises: Why does heating of enamel, whether through oven treatment or specific laser protocols, improve its acid resistance? Current theories suggest that the increased temperatures induce crystallographic alterations and decompose the organic matrix, thereby reducing the diffusion of ions. Subjecting enamel to heat causes several alterations in its composition and structure, especially in the hydroxyapatite phase. These alterations, which vary with temperature, directly impact enamel solubility and include decreased water content, protein breakdown, carbonates elimination, pyrophosphates formation, and crystals growth^16,18,39^. In addition, pyrophosphate formation occurs within the temperature range of 100–650 °C, a transformation known to substantially decrease enamel solubility^16^.

It is important to note that Palamara et al. (198^40^ reported ultrastructural changes at 200–600 °C, including increased intra‑ and inter‑crystalline voids and the appearance of β‑TCP crystals from 400 °C onwards. These changes were interpreted as potentially increasing enamel solubility; however, that study did not directly assess solubility or erosion resistance. In contrast, the present study evaluated enamel behavior under dynamic erosive cycling, which represents a different outcome measure. Thus, the ultrastructural observations of Palamara et al. complement, rather than contradict, the reduced surface loss observed here at 300–500 °C.

Taken together, these thermally induced modifications—reduced water content, altered crystal chemistry, pyrophosphate formation, and microstructural reorganization provide a plausible explanation for the marked increase in erosion resistance observed in the present study.

In addition, thermal exposure is known to influence the water content of dental enamel, and this may partially explain the changes observed in our study. Enamel contains two main water compartments: free water located within interprismatic pores, and structural water incorporated within the hydroxyapatite lattice. These compartments differ markedly in their thermal behavior. Free water is removed at relatively low temperatures and contributes to higher diffusion rates for exogenous ions, whereas structural water requires substantially greater energy for release^41^.The preferential loss of free water during heating may therefore reduce enamel porosity and permeability, potentially limiting ion diffusion and, consequently, decreasing enamel susceptibility to erosive dissolution^40^. However, this interpretation remains speculative and should be confirmed by studies directly assessing changes in enamel water content and transport properties after thermal exposure. The present study showed that heating enamel to 300 °C and 500 °C significantly enhanced its resistance to erosion, supporting the theory that several structural modifications are likely key mechanisms underlying the observed protective effects.

Tin-containing fluoride solutions are currently the gold standard for erosion prevention, with proven effects in vitro^11^ and in vivo^12^. Their protective mechanism involves forming a tin-rich layer on enamel, improving acid resistance and reducing mineral loss^42^. A 4-year randomized controlled trial^12^ further validated the efficacy of the combination of a mouth rinse and toothpaste, both products containing stannous fluoride (SnCl₂), amine fluoride (AmF), and sodium fluoride (NaF) in preventing dental erosion. The tin containing oral health products significantly reduced enamel erosion when compared to the non-tin but fluoride containing control group. However, it also increased tooth discoloration, while no notable changes in saliva pH or dentin hypersensitivity were observed. Despite this, the present study showed that certain heating temperatures resulted in a significantly greater reduction in enamel surface loss compared to fluoride treatment. This highlights the potential of temperature-induced changes in enamel as a key factor in improving prevention strategies as discussed above.

In summary, this study is among the first to establish a direct, quantitative relationship between heating temperature and enamel erosion resistance, with the most pronounced protective effect, an 87% reduction in surface loss observed at 500 °C. While oven heating itself is not clinically applicable, the findings provide mechanistic insights into temperature-driven structural modifications that can guide the optimization of CO₂ laser protocols. Importantly, the identification of a protective temperature range (300–500 °C) provides a concrete target for future laser-based interventions. Future research should focus on optimizing CO₂ laser parameters to maintain enamel surface temperatures within the protective range, supported by real-time monitoring systems that safeguard dental tissues against overheating. At the same time, exploring synergistic effects with low-concentration fluoride could allow similar protective benefits to be achieved at lower thermal thresholds. Together, these advances hold strong translational potential for developing minimally invasive, office-based preventive treatments.

A limitation of the present study was the utilization of bovine enamel only, which may not precisely replicate the characteristics of human enamel. While bovine enamel has a similar composition and optical qualities to human enamel, it is often more porous, has slightly higher carbonate levels, and exhibits more surface loss when exposed to erosive and erosive-abrasive conditions^43^. Moreover, the in vitro design also lacks critical biological factors such as saliva, enzymatic activity, and mechanical forces like mastication, reducing clinical relevance. Furthermore, prolonged oven heating differs from the short (fractions of a second), localized exposure of CO₂ lasers, complicating direct comparisons. Additionally, the study focused solely on citric acid erosion, ignoring other real-world factors like abrasion, attrition, or acquired-pellicle presence. These limitations necessitate caution when extrapolating the findings of the present study to human dental tissues.

## Conclusion

Controlled heating of enamel to 100 °C and 500 °C significantly increased enamel erosion resistance. Enamel heated to 300 °C, 400 °C, and 500 °C outperformed both no treatment and daily treatment with tin-containing fluoride solution in vitro. The highest percentual reduction relative to negative control was observed by the 400 °C and 500 °C groups and was of 77% and 87% respectively, while tin-containing fluoride treatment reduced enamel surface loss by 50%. While oven heating provided standardized conditions, its clinical relevance is limited but rather contribute to a better understanding of the erosion protective effect of the CO_2_ laser irradiation in future studies.

## Supplementary Information

Supplementary Information.

## Data Availability

Raw data that support the findings of this study is available from the corresponding author, upon reasonable request.
