# Supplementary material for: Controlled heating between 100 and 500 degrees celsius improves enamel resistance to erosion in vitro
Source: Sci Rep. 2026 Apr 9;16:12032. doi: 10.1038/s41598-026-47191-5 (PMC13069023; doi:10.1038/s41598-026-47191-5)
Supplement: Supplementary file 1 — Supplementary Information. [file 41598_2026_47191_MOESM1_ESM.docx]

**Supplementary Material**

**Controlled Heating Enhances Enamel Resistance to Erosion: An *In Vitro* Study at the 100 to 500 °C temperature range**

Richard J. Wierichs^a,b^ ^†^, Seyed Ahmad Banihashem Rad^b,c^ **^†^**, Joel Glöckler^d^, Marin D. Bilandzic^d^, Stephanie Garofalo ^d^, Gabriella T. Schrotter ^f^, Samira H. Niemeyer^b^, Guglielmo Campus^g^, Hendrik Meyer-Lueckel^b^, Marcella Esteves-Oliveira * ^d^

Content

[1. Pilot Test Oven Heating 2](#_Toc174964673)

[2. Histological Results Pilot Study 3](#_Toc174964674)

# **Pilot Test Oven Heating**

The two heating rates 1 K/min (=1°C/min) and 5 K/min (=5°C/min) of the oven are showed in Fig. 1.

***Supp. Mat. Figure 1.***

*Heating curve showing the temperature in °C (y-axis) and time in minute (x-axis): For enamel sample heated at heating rate of 5K/min, the holding time was 5 minutes and total time in the oven: 570 minutes (heating rate: 5  K/min. = 5 °C/min.) For the enamel sample heated at the heating rare of 1 K/min, the holding time was 1 minute and total time in the oven: 880 minutes (heating rate: 1 K/min.=1°C/min.).*

# **Histological Results Pilot Study**

The pilot study showed that the samples underwent changes in shape and color due to the oven heating. Specimens at higher temperature levels showed increased cracking and fracturing (**Figure 2**).


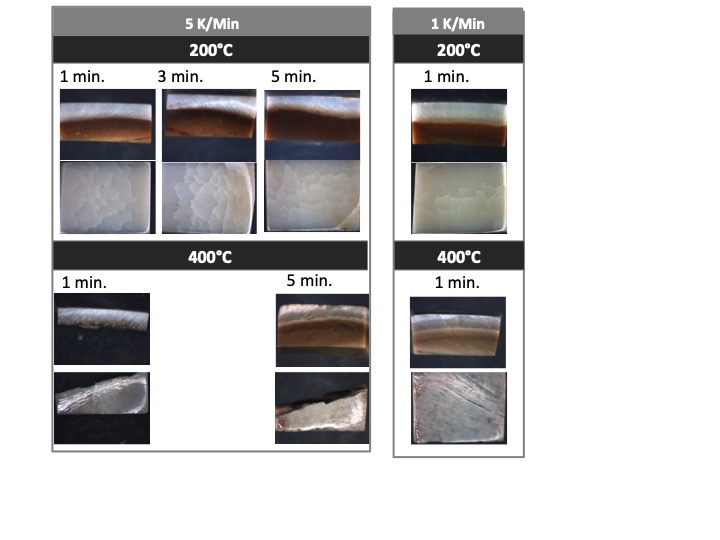


**Supp. Mat. Fig. 2.** Surface and cross-sectional images of the dental samples heated with different heating rates (5 K/min or 1 K/min) and with different holding times from 1, 3 and 5 min. Samples treated with a lower heating rate showed lower fracture values. Significantly more samples in the 400°C group with a heating rate of 5 K/min cracked than in the comparison group heated with only 1 K/min. It was also evident that more samples were damaged with increasing holding time. Consequently, in the main experiment, the heating rate was chosen to be 1 K/min and the holding time was set to one minute. In order for fewer samples to suffer mechanical damage, it was decided to remove the dentin from the samples in the 500°C group beforehand.
